# Diagnostic Accuracy and Concordance of Standardized vs. Non-Standardized Joint Physical Examination for Assessing Disease Activity in Rheumatoid Arthritis: A Paired Comparison Using Ultrasound as Reference Standard

**DOI:** 10.3390/jcm14155334

**Published:** 2025-07-29

**Authors:** Yimy F. Medina, Martin A. Rondón

**Affiliations:** 1 PhD Program in Clinical Epidemiology, Department of Clinical Epidemiology and Biostatistics, Pontificia Universidad Javeriana, Bogotá 110311, Colombia; martin.rondon@javeriana.edu.co; 2 Rheumatology Unit, Internal Medicine, Universidad Nacional de Colombia, Bogotá 111321, Colombia; 3 Department of Clinical Epidemiology and Biostatistics, Pontificia Universidad Javeriana, Bogotá 110311, Colombia

**Keywords:** rheumatoid arthritis, joint physical examination, musculoskeletal ultrasound, diagnostic accuracy, concordance, disease activity, flare

## Abstract

**Objective:** Physical joint examination is fundamental in rheumatoid arthritis (RA) assessment. This study evaluated the diagnostic accuracy and agreement between standardized and non-standardized physical joint examinations in RA patients using musculoskeletal ultrasound as the reference standard. **Methods:** We assessed the joints for tenderness and swelling, calculating sensitivity, specificity, and predictive values. Musculoskeletal ultrasound was used as the reference standard, with adjustment for imperfect reference bias. Agreement between the methods was evaluated using the average kappa coefficient. **Results:** A total of 1496 joints were evaluated. Without adjustment for imperfect reference bias, standardized examination showed higher sensitivity for detecting pain and swelling than non-standardized examination. Specificity was similar for pain but higher for swelling in standardized examination. After bias adjustment, standardized examination sensitivity improved for pain (93.8% vs. 77.3%; 95% CI: 0.14–0.19) and swelling (91.9% vs. 60.0%; 95% CI: 0.29–0.34). Tenderness specificity remained comparable (standardized examination: 75.4%, non-standardized examination: 76.3%), while the non-standardized examination maintained superior swelling specificity (85.7% vs. 77.1%). Standardized joint examination demonstrated significantly higher concordance than non-standardized assessment in evaluating joint tenderness; standardized assessment yielded significantly greater average kappa coefficients under both false-positive-prioritized (0.44 vs. 0.37; *p* = 0.01) and false-negative-prioritized scenarios (0.59 vs. 0.45; *p* < 0.0001). For joint swelling, standardized evaluation showed significantly higher concordance when false negatives were considered more critical (0.59 vs. 0.37; *p* < 0.0001), whereas differences under false-positive prioritization were not statistically significant. **Conclusions:** Standardization of the physical joint examination significantly improves diagnostic accuracy and agreement in detecting joint tenderness and swelling in patients with rheumatoid arthritis. Implementing a standardized physical examination protocol may enhance disease activity diagnosis and optimize clinical management of RA.

## 1. Introduction

Rheumatoid arthritis (RA) is a chronic, systemic, multifactorial autoimmune disease characterized by inflammation and joint destruction [1]. RA activity is clinically defined on physical examination by joint tenderness and swelling in individual patients [2,3]. However, this evaluation has limitations—it is not standardized, leading to significant variability and low reproducibility, which in turn causes discrepancies among evaluators during patient follow-up [4,5].

A systematic literature review concluded that although clinical assessment is fundamental for diagnosing and monitoring patients with RA, evidence on performing joint examinations remains scarce and lacks standardization [6]. Studies conducted across multiple countries highlight the absence of a unified technique for joint examination in patients with RA that could be taught to healthcare providers [7,8,9,10,11].

A national survey conducted in Colombia in 2018 among rheumatologists revealed notable variability in concepts and approaches related to joint physical examination in patients with RA [12]. Recently, evidence-based consensus methods defined the essential components of physical examination in RA, resulting in the development of a standardized joint physical examination protocol [13].

In this study, we compared the diagnostic accuracy and concordance between the standardized joint physical examination and a non-standardized physical examination in patients with RA, using joint ultrasonography as the reference standard.

## 2. Materials and Methods

Given that the primary objective of this study was to evaluate the diagnostic accuracy and interobserver agreement of joint-level examination techniques, the unit of analysis was the individual joint, not the patient. This approach is consistent with previous methodological frameworks in rheumatology, where joint-based assessment is central to disease activity measurement and physical examination validation [6,14,15].

### 2.1. Design

This paired diagnostic test study compared two methods of joint assessment in patients with RA: the standardized examination (SE) versus the non-standardized examination (NSE), using joint ultrasonography (US) as the reference standard. Although ultrasonography is not a definitive reference standard, its diagnostic performance is comparable to that of magnetic resonance imaging, which is considered a validated clinical reference standard. Both methods significantly correlated with histopathological findings, which are the gold standard for evaluating synovial disorders in RA [16,17].

### 2.2. Population

The study population included patients aged >18 years. All patients included in the study fulfilled the 2010 American College of Rheumatology/European League Against Rheumatism (ACR/EULAR) classification criteria for RA, as assessed by rheumatologists prior to inclusion [18], with no restrictions regarding disease activity level. The overall disease activity was assessed using the Disease Activity Score in 28 joints (DAS28) [19], incorporating erythrocyte sedimentation rate, 28-swollen joint count, 28-tender joint count, and the patient’s global assessment. However, DAS28 values were not used for inclusion or stratification purposes in the present analysis. Instead, for the purpose of this study, each joint was individually evaluated and recorded as either positive or negative for swelling and tenderness based on physical examination findings, regardless of the overall DAS28 score. This allowed for a joint-level comparison of examination methods against musculoskeletal ultrasound findings.

Patients were consecutively enrolled from various rheumatology care centers in Bogotá, Colombia, at distinct stages of disease progression.

### 2.3. Intervention

The standardized joint physical examination refers to a structured and rigorously developed protocol previously established and validated by our research group using consensus methodology (RAND/UCLA Appropriateness Method), as detailed in prior publications [13,20]. This protocol includes explicit, operationalized definitions for the inspection, palpation, and specific maneuvers of each joint, ensuring a uniform and systematic approach to identifying clinical signs of synovitis such as tenderness and swelling. The examination follows a predefined sequence and standardized instructions to minimize interobserver variability, enhance reproducibility, and facilitate a comprehensive assessment of joint involvement in patients with rheumatoid arthritis.

In contrast, the non-standardized joint examination reflects routine clinical practice, wherein rheumatologists assess joints based on their individual clinical experience, judgment, and customary techniques, without adherence to any formalized protocol or checklist. This approach inherently permits variability in examination methods, documentation, and interpretation, thereby representing the heterogeneity and pragmatism typical of real-world clinical environments.

Ultrasonography was used as a reference standard to identify signs of synovitis or joint inflammation following the recommendations established by the European League Against Rheumatism (EULAR) [21]. 

Ultrasound examination process (reference standard): During the ultrasound scanning and image acquisition procedures, which included patient and joint positioning, anatomical region positioning, scanned surfaces (e.g., volar, dorsal), and transducer positioning (e.g., transverse, longitudinal, and static examination), we followed the recommendations established by the EULAR for this field [21]. We performed ultrasound evaluations with a SonoScape device (SonoScape Colombia S.A.S., Carrera 15 #88-64, Office 601, Bogotá D.C., Colombia; www.sonoscape.com.co). The specific model used was the SonoScape X5, equipped with a high-frequency linear array transducer (L741, 4–18 MHz). The ultrasound system operated with software version V1.3.2. Equipment calibration and maintenance were conducted according to the manufacturer’s guidelines. Employing a high-frequency linear transducer (L743, 6–18 MHz) for small and superficial joints (interphalangeal, metacarpophalangeal, wrist, and ankle), and a mid-range linear transducer (L741, 5–14 MHz) for larger and deeper joints (knee, hip, and shoulder). We did not modify the equipment or software during the study. We used grayscale modalities (frequency: 10–18 MHz, optimized gain and field depth) and color Doppler (frequency: 6–12 MHz, wall filter: 50–100 Hz, pulse repetition frequency: 500–1000 Hz), following parameters validated in the literature for musculoskeletal evaluation, with adjustments aimed at optimizing flow visualization and minimizing artifacts [21]. We considered a joint normal by ultrasound if it did not present hypoechoic images suggestive of synovial hypertrophy and lacked Doppler signal in the synovium. We defined ultrasound synovitis according to the criteria of the combined ultrasound index, which considers the presence of synovial hypertrophy in grayscale at least of grade 1, together with a Doppler signal of at least grade 1, as positive. We based this evaluation on the EULAR-OMERACT composite scale for synovitis, which classifies synovitis on a scale of 0 to 3 [22,23]. The sonographer interpreted the ultrasound images concomitantly to their acquisition. To ensure compliance with ethical and quality standards, we thoroughly reviewed the images included in the manuscript. We verified the removal of any information that could compromise patient confidentiality, and we clearly labeled structures of interest to facilitate their interpretation. The selected images maintained consistency with the content of the manuscript and we optimized them to ensure adequate visualization of relevant ultrasound findings [24].

Musculoskeletal ultrasound examinations were conducted by a board-certified rheumatologist with over 30 years of clinical experience and more than 15 years of dedicated expertise in musculoskeletal ultrasonography. The examiner holds advanced certification in ultrasound from the European League Against Rheumatism (EULAR) and has served as a faculty member and evaluator in EULAR-endorsed ultrasound training courses. Although intra- or interobserver reliability was not directly assessed in this study, the examiner’s diagnostic performance has been previously validated in international collaborative studies, including the OMERACT Calcium Pyrophosphate Deposition Disease Ultrasound Subtask Force. In this multicenter reliability exercise, involving an extended set of joints and the application of standardized definitions, the interobserver agreement for detecting sonographic signs of CPPD achieved substantial to almost perfect kappa values (κ = 0.75–0.88), confirming the examiner’s consistency and reliability under standardized conditions [25].

To ensure consistency across assessments, all ultrasound examinations were conducted by a single rheumatologist with extensive musculoskeletal ultrasound training, following standardized scanning procedures as recommended by EULAR [21]. While formal intra- or interobserver reproducibility statistics were not collected, the exclusive use of one expert operator aimed to reduce interoperator variability and maintain internal consistency. This design choice reflects common practice in exploratory diagnostic accuracy studies and helps isolate methodological comparisons from operator-related variation [26].

In this study, signs of synovitis or joint inflammation were defined as the presence of synovial hypertrophy, characterized by a hypoechoic thickening of the synovial membrane, concomitant with an increase in blood flow (synovial hyperemia), evaluated using Doppler ultrasonography in the joint cavity, employing ultrasonography techniques that included grayscale mode (B-mode) and Doppler (color, power, and pulse) [21].

Data collection was performed prospectively, ensuring that the SE, NSE, and US tests were performed on the same day. A total of 68 joints per patient were explored to evaluate tenderness, and 66 of the same joints were assessed for swelling, excluding the hips for the latter evaluation.

The principal investigator, a rheumatologist familiar with the method, performed the standardized examination [13]. Another rheumatologist, selected randomly through a call by the Colombian Association of Rheumatology, who was unfamiliar with the standardized examination, performed the non-standardized examination according to their usual clinical practice. The rheumatologists who performed the standardized and non-standardized examinations were experienced professionals with at least 5 years of experience and daily practice in managing patients with RA to ensure that any observed differences in results were due to the test methodology and not their level of expertise. Joint ultrasonography was performed by a different rheumatologist, with 30 years of experience in rheumatology and 15 years of experience in joint ultrasonography, with advanced training and certification in ultrasonography granted by the European League Against Rheumatism (EULAR).

To minimize the risk of bias in evaluation, no examiner knew the results of their colleagues’ evaluations or patients’ clinical data. The variable of interest, which was the presence of clinical findings in joint evaluation, was binary, with each test being classified as positive or negative for both tenderness and swelling. Similarly, synovitis on ultrasonography was evaluated as binary, with the same classification. A positive result was defined as the concomitant presence of joint tenderness and swelling during the clinical examination, thereby confirming the association between ultrasonographic findings and clinical evaluation.

### 2.4. Data Analysis

Each joint was considered the unit of analysis with equal weighting. We compared the sensitivity and specificity for diagnostic studies with binary variables to assess the accuracy of the standardized and non-standardized examinations against ultrasonography. We followed the recommendations of Roldán-Nofuentes et al. Therefore, we used the Wald test for low prevalences (5–10%) and small sample sizes (*n* = 50). For moderate sample sizes (*n* = 100), we applied Bonferroni or Holm corrections. For larger samples (100 ≤ *n* ≤ 1000), we used the McNemar test with continuity correction. We applied all tests to all prevalences to observe differences in the results, ensuring an exhaustive analysis [27].

To compare the positive predictive values (PPVs) and negative predictive values (NPVs) of the two paired diagnostic tests, the null hypothesis of equality was evaluated using the Wald test. The generalized weighted score test by Kosinski, adjusted with Holm’s method, was applied to control for type I errors [28]. Additionally, recognizing that not all cases of disagreement carry the same clinical implications, classification errors were weighted according to their clinical relevance. For this purpose, the average kappa coefficient was used to evaluate the agreement between the diagnostic tests, taking these errors into account, and was applied in two distinct scenarios: (1) When false positives had more severe clinical consequences, and (2) when false negatives were the primary concern [29]. To formally compare the average kappa coefficients between the standardized and non-standardized physical examinations, hypothesis tests were conducted under both weighting conditions (false positives or false negatives). Standard errors of the kappa coefficients were estimated to construct 95% confidence intervals for the differences. Statistical significance was assessed using two-sided tests with a significance level set at α = 0.05. All calculations were based on the analytical framework for paired binary tests under differential weighting schemes, depending on whether false positives or false negatives were considered more clinically relevant [29]. The interpretation of the obtained values followed the criteria established by Landis and Koch [30].

Sample size determination addressed both diagnostic accuracy and agreement objectives. For the diagnostic accuracy component (sensitivity, specificity, and predictive values), we employed a paired design in which both the standardized and non-standardized examinations were applied to the same subjects, resulting in non-independent observations. The sample size was estimated using the McNemar test framework, focusing on discordant pairs, as recommended for paired diagnostic test studies. The parameters for the proportion of discordant pairs were derived from previous work by Terslev et al. [31], which compared ultrasound and clinical examination in rheumatoid arthritis. Based on their findings—π discordant = 0.20 for joints without pain and swelling (ratio Ψ = 6.66) and π discordant = 0.23 for joints with pain and swelling (ratio Ψ = 2.33)—and using the Stata 14.0 software with 80% power and 5% type I error rate, we calculated a minimum requirement of 240 joints for the diagnostic accuracy analysis [32].

For the agreement analysis, we calculated the sample size needed to estimate the interobserver agreement using the kappa coefficient, assuming an expected kappa of 0.8, a 95% confidence level, and a maximum confidence interval width of 0.1. The sample size formula incorporated the expected kappa, the desired precision, and the estimated proportion of disagreement, as outlined by Machin et al. [33]. This calculation indicated a minimum of 554 joints was required for agreement analysis.

To ensure sufficient statistical power and precision for both study objectives, we adopted the larger of the two calculated sample sizes, setting the minimum at 554 joints. To further enhance robustness and reduce estimate variability, we doubled this figure to 1108 joints, corresponding to approximately 17–18 patients, based on the 68-joint assessment protocol per patient. Ultimately, our study included 22 patients and evaluated 1496 joints, exceeding the minimum requirements for both diagnostic accuracy and agreement analyses. This approach ensured that the study was adequately powered to provide accurate and precise estimates for all outcomes [33].

Articular ultrasonography is an imperfect reference standard, and its performance can be influenced by equipment quality and operator experience [34,35,36]. To correct this bias caused by an imperfect reference standard, we adjusted the estimates according to the methodology of Reitsma and Staquet [37,38]. This adjustment was based on the findings of studies comparing musculoskeletal ultrasound and magnetic resonance imaging in patients with RA [39,40,41,42].

All statistical analyses were performed using the R software (version 4.3.2; R Foundation for Statistical Computing, Vienna, Austria), a widely used open-source environment for statistical computing.

## 3. Results

The study analyzed 1496 joints from 22 patients (5 men and 17 women). The mean disease duration was 4.3 years (standard deviation 2.2), ranging from 1 to 9 years. The mean age was 41 years (standard deviation 7.8). Attending rheumatologists classified disease severity as mild in eight patients (36.36%), moderate in eight (36.36%), and severe in six (27.7%). Ultrasonography was performed on all 1496 joints; however, deformities prevented the evaluation of 33 joints, leaving 1463 joints examined. Standardized and non-standardized examinations were applied to assess tenderness in these joints. For the evaluation of swelling, researchers examined 1419 joints, excluding hips because of their inaccessibility to palpation. 

### 3.1. Sensitivity and Specificity

The sensitivity and specificity results for detecting joint tenderness are presented in Table 1. The standardized examination showed higher sensitivity than the non-standardized examination for identifying joint tenderness. No statistically significant differences were observed in specificity between the two methods for joint tenderness. These findings were consistent with the results of both individual and global statistical tests.

For joint swelling evaluation, the standardized examination showed higher sensitivity than the non-standardized examination, whereas the non-standardized examination exhibited higher specificity (Table 2). The observed differences in sensitivity and specificity between the two examinations remained significant when applying individual and global statistical tests.

### 3.2. Predictive Values

In evaluating joint tenderness under low-prevalence scenarios (10%), such as those occurring in primary care or general outpatient settings, researchers found no significant differences in PPV or NPV between the standardized examination and non-standardized examination methods. Kosinski and Holm analyses confirmed this diagnostic equivalence. In scenarios with intermediate prevalences (20–50%), typical of general rheumatology consultations, the standardized examination showed a higher NPV than the non-standardized examination, with no differences identified in PPV. Under high-prevalence conditions (90%), such as those in specialized rheumatology clinics, no differences were identified in PPV, but the standardized examination showed a higher NPV, as validated by the Kosinski and Holm tests (Table 3).

For evaluating joint swelling under low-prevalence conditions (10%), researchers detected no significant differences in PPV but did find differences in NPV, where the standardized examination performed better. In situations with intermediate prevalences (20–50%), PPVs were comparable between both methods, and better NPV results were observed for the non-standardized examination. In high-prevalence contexts (90%), both methods achieved high PPVs without significant differences, but the standardized examination maintained a higher NPV (Table 4).

### 3.3. Correction to Imperfect Reference Bias

After applying adjustments for the bias derived from the imperfect reference standard (US), the researchers found that, when comparing the standardized examination and non-standardized examination, the standardized examination maintained a higher sensitivity for detecting tenderness and swelling, whereas the non-standardized examination showed a higher specificity for swelling. These differences were statistically significant. Regarding specificity for tenderness, no statistically significant differences were observed between the two examinations, despite an increase after correcting for this bias (Table 1 and Table 2).

After adjustment for reference bias, the predictive values indicated that the standardized examination had a greater ability to correctly predict the presence of joint tenderness and swelling, with statistically significant differences in the observed proportions. For NPV, both examinations showed improvements in correctly ruling out these conditions, with superior performance in the standardized examination and statistically significant differences (Table 5).

### 3.4. Concordance

For joint tenderness, when false positives were considered more clinically detrimental, the standardized examination demonstrated a significantly higher average kappa coefficient (0.44, SE = 0.022) compared with the non-standardized examination (0.372, SE = 0.024). This difference was statistically significant (*p* = 0.01), with a 95% confidence interval of 0.01–0.12, leading to the rejection of the null hypothesis of equal concordance. Conversely, when false negatives were regarded as more critical, the standardized examination again yielded a substantially higher average kappa (0.59, SE = 0.023) relative to the non-standardized method (0.45, SE = 0.027), with this difference being highly significant (*p* < 0.0001) and a 95% confidence interval ranging from 0.07 to 0.21. Regarding joint swelling, in scenarios where false positives were prioritized, the average kappa for the standardized examination was 0.457 (SE = 0.023) compared with 0.40 (SE = 0.028) for the non-standardized examination; however, this difference did not reach statistical significance (*p* = 0.08), as the 95% confidence interval (−0.007 to 0.112) included zero. In contrast, when false negatives were considered more important, the standardized examination showed a markedly higher average kappa (0.59, SE = 0.024) than the non-standardized examination (0.37, SE = 0.027), a difference that was highly significant (*p* < 0.0001), with a 95% confidence interval between 0.160 and 0.279.

## 4. Discussion

This study compared the diagnostic accuracy and agreement between standardized and non-standardized joint examinations in patients with rheumatoid arthritis (RA), using musculoskeletal ultrasound as the reference standard, with adjustment for imperfect reference standard bias. Rheumatoid arthritis (RA) requires consistent and accurate joint evaluation, as joint counts are integral to disease activity scoring and therapeutic decisions [43]. However, variability in clinical joint examination remains a concern due to a lack of a standardized methodology [13,15,44]. Our findings reinforce the benefit of using a standardized examination technique, which demonstrated improved sensitivity for joint tenderness and swelling, reducing false negatives—a key factor in early RA management [5]. This method also yielded higher negative predictive values (NPVs) in intermediate- and high-prevalence scenarios, making it particularly useful for ruling out active inflammation in patients with significant disease burden. Although specificity was slightly lower, the trade-off favored clinical utility in detecting active disease.

Moreover, concordance analyses showed higher kappa values for the standardized method, especially in scenarios prioritizing the avoidance of false negatives. This aligns with prior evidence that standardized protocols reduce subjective variability and improve diagnostic reliability [11,12,44]. For joint pain and swelling, kappa values were consistently higher with the standardized approach, although for swelling, the difference did not reach statistical significance when false positives were prioritized—likely due to the inherent subjectivity in assessing swelling [39]. These findings emphasize the importance of structured clinical training and suggest the standardized method’s potential for broader implementation. Further validation in diverse populations is warranted to assess generalizability and inform clinical guideline integration [15].

Previous attempts have been made to standardize the physical examination. Almoallim et al. attempted to standardize the physical examination of 22 joints of the hands and wrists in RA [45]. In terms of sensitivity and specificity, the results varied depending on the technique used, with sensitivity for the “scissor” method for detecting swelling in the MCP ranging between 66% and 74%, while the “2-thumb” method achieved a sensitivity of 80% in the wrist. No specific information was provided, nor was there a comprehensive comparative analysis of other techniques or similar studies [45].

Unlike Almoallim et al., our study expanded the scope of the physical examination to 68 synovial joints, including body regions beyond the hands and wrists [41,42,43]. In addition, we implemented an explicit standardization process in which recommendations derived from a national consensus of expert rheumatologists were adopted and elaborated using the modified RAND-UCLA methodology [46]. This methodology combined a systematic literature review with rounds of evaluation and discussion among experienced clinicians to define appropriate techniques for joint exploration in RA. As a result, we established a set of specific recommendations intended to homogenize the clinical approach to joint assessment, standardizing exploration maneuvers for the identification of clinical signs of tenderness and swelling. Thus, our study represents a significant methodological advancement compared to the limited prior attempts mentioned in the literature, which were constrained by their narrow scope and lack of explicit standardization. The results of this study suggest that our methodology offers a robust tool for the clinical evaluation of inflammatory activity in RA, contributing to the development of more accurate and applicable diagnostic standards.

Although articular histology is the ideal gold standard, its application in clinical practice is unfeasible due to its invasive nature and associated risks [47]. MRI is considered a valuable clinical reference standard, but its cost and time-consuming nature limit its accessibility in routine clinical settings [48]. Ultrasonography has emerged as a crucial tool for real-time assessment of synovial alterations, both structural and vascular, establishing itself as an accessible and effective alternative in clinical practice [49]. While musculoskeletal US is widely recognized as a reasonable and accessible reference standard for the assessment of synovitis in rheumatoid arthritis, it is important to acknowledge its inherent operator and machine dependence. The diagnostic performance of US can be influenced by the examiner’s expertise, the calibration and technical specifications of the equipment, and the standardization of scanning protocols. Although our study followed established EULAR guidelines for image acquisition and interpretation, and the US assessments were performed by a highly experienced rheumatologist using validated protocols, these factors may limit the reproducibility of our findings in settings with less experienced operators or different equipment. Furthermore, variability in US performance across centers and practitioners has been documented in the literature, underscoring the need for careful consideration of these limitations when interpreting our results and generalizing them to broader clinical practice [50,51].

Although musculoskeletal ultrasound benefits from established internal standardization protocols that enhance its accuracy and reproducibility, it is important to note that US is not currently included in the official diagnostic criteria or definitions of disease activity for RA. Widely accepted classification criteria, such as the 2010 ACR/EULAR criteria [18], and composite disease activity indices like DAS28 [19], do not incorporate ultrasound findings as standardized parameters. Therefore, while US serves as a valuable complementary tool for detecting synovitis and guiding clinical decisions, its integration into formal RA diagnostic and activity frameworks remains under evaluation.

Regarding the effects of biases, it is important to consider that operator experience can influence the interpretation of results in both physical examination and ultrasonography. Variability in physical-examination techniques can affect the reproducibility of clinical findings, underscoring the need for standardized protocols to minimize these biases [48]. The detection of synovitis by ultrasonography underscores the relevance of this diagnostic modality in patients with RA [52]. Correcting for bias associated with the suboptimal reference standard (ultrasonography) allowed a more accurate estimation of the diagnostic capability of both tests. Although we acknowledge that both ultrasound and physical examination evaluate synovitis in real time and the paired design may introduce some degree of dependent misclassification, all assessments in our study were performed independently and in a blinded manner, with examiners unaware of the patients’ clinical characteristics and the results of other evaluators; these measures were implemented to minimize shared biases and strengthen the validity and interpretability of our findings.

Our study has some limitations. First, the dependence on operator experience, both in the execution of the physical examination and in ultrasonography, could have introduced biases in the interpretation of results. These biases may have influenced the overestimation or underestimation in both clinical findings and imaging. Greater operator experience in clinical assessment could have increased the detection of joint signs [53,54], thereby improving the sensitivity of physical examination. Conversely, greater operator experience in performing ultrasonography could have resulted in greater detection of synovitis, which would generate a lower sensitivity of the physical examination compared with ultrasonography. A notable limitation of this study is the operator dependence of musculoskeletal ultrasound. All US assessments were conducted by a single expert rheumatologist with advanced musculoskeletal ultrasonography training to ensure internal consistency. While this approach minimized interobserver variability and enhanced the reliability of joint-level comparisons, it may limit reproducibility in clinical environments with less experienced personnel. The diagnostic accuracy of US can vary substantially depending on the operator’s skill and familiarity with joint-specific pathology, as demonstrated in previous comparative studies. Therefore, the applicability of these findings in general practice settings may be constrained unless similar levels of training and standardization are ensured.

In addition to its known operator dependence, the diagnostic performance of musculoskeletal ultrasound is also highly influenced by the quality and technical specifications of the equipment used. Modern, high-frequency probes and advanced imaging software can significantly enhance the visualization of synovial pathology, thereby improving the detection of synovitis.

Second, the performance of both the standardized and non-standardized examinations was evaluated by a single rheumatologist for each assessment. Although this approach aimed to minimize interobserver variability, it may not fully capture the heterogeneity inherent in routine clinical practice. While we sought to mitigate this limitation by ensuring that both rheumatologists had experience in managing patients with RA, we cannot entirely rule out that the observed differences might stem from individual examiner characteristics rather than the examination methodology alone. Third, we did not evaluate the impact of standardization on long-term clinical practice, for which follow-up studies would be necessary. Finally, the adjustment for bias from the imperfect reference standard improved the analysis but did not eliminate the inherent limitations of using an imperfect standard. In the future, it will be important to validate these findings in multicenter studies with greater population heterogeneity and a greater number of operators to reinforce clinical applicability. Histopathology is considered the gold standard for evaluating synovitis. However, its application is limited because of its invasive nature and logistical difficulties. This discrepancy between the ideal standard (histopathology) and clinically practical methods (ultrasonography or magnetic resonance imaging) underscores the need for innovative methodological designs that reconcile diagnostic validity with practical feasibility.

We acknowledge that, although each assessment was performed by a different experienced rheumatologist to preserve methodological integrity and prevent cross-contamination of techniques, some residual confounding between examiner and technique cannot be entirely excluded. Future studies employing crossover or multi-examiner designs are warranted to further disentangle the effects of technique from examiner variability. Also, future research with larger and more diverse patient samples could explore whether the comparative performance of standardized and non-standardized examinations varies by disease activity, duration, or demographic factors.

A methodological limitation of this study is that joints were analyzed as independent units, without adjustment for clustering within patients. This approach was selected to reflect the joint-level diagnostic focus of the research; however, it may have resulted in overestimation of statistical precision due to the non-independence of joint observations. Similar strategies have been used in prior studies assessing joint-level agreement or diagnostic performance in rheumatoid arthritis [55,56,57].

It is also important to note that our study was designed to assess diagnostic accuracy at the joint level, rather than to compare patient-level disease activity categories between examination methods. As such, we did not aggregate joint findings to classify patients according to overall disease activity (e.g., high, moderate, low, or remission). This joint-centric approach aligns with our primary objective of evaluating the performance of physical examination in detecting pathology in individual joints and is consistent with prior studies in the field.

## 5. Conclusions

This study demonstrates that standardizing the physical joint examination, as implemented through the Standardized Physical Articular Examination protocol, significantly improves the sensitivity and diagnostic accuracy for detecting joint tenderness and swelling in patients with rheumatoid arthritis, compared to non-standardized assessment. This enhancement in diagnostic performance has important clinical implications, as it may facilitate earlier and more accurate diagnosis, leading to improved management of RA.

Moreover, the higher negative predictive value of the standardized examination in intermediate- and high-prevalence settings underscores its utility in confidently ruling out active inflammation and potentially reducing unnecessary diagnostic procedures.

Overall, these findings highlight the critical importance of adopting a standardized physical joint examination approach in clinical practice to optimize diagnostic accuracy and improve patient outcomes in rheumatoid arthritis. However, multicenter studies involving more diverse patient cohorts and clinical environments are warranted to validate these results and to determine the extent to which standardized joint examination protocols can be integrated into routine practice across different settings.

## Figures and Tables

**Table 1:** Sensitivity and specificity results of SE and NSE—joint tenderness; 1463 joints.

	Standardized Exam (SE)			Non-Standardized Exam (NSE)		
	Positive	Negative	Total	Positive	Negative	Total
Ultrasound Positive	256	115	442	52	19	442
Ultrasound Negative	114	907	1021	148	873	1021
Total	370	278	1463	200	615	1463
Diagnostic Accuracy—Before and After Adjustment for Reference Standard Bias						
Parameter	Standardized Exam (%)			Non-Standardized Exam (%)		95% CI for Difference
Sensitivity	83.9 (95% CI: 80.2–87.0) → 93.8 (adjusted)			69.6 (95% CI: 65.2–73.7) → 77.3 (adjusted)		(0.14; 0.19)
Specificity	72.8 (95% CI: 70.0–75.5) → 75.4 (adjusted)			74.3 (95% CI: 71.5–76.9) → 76.3 (adjusted)		(–0.03; 0.02)
Statistical Comparison (SE vs. NSE) Before Adjustment for Reference Standard Bias						
- Sensitivity: McNemar test χ^2^ = 23.01, *p* < 0.001 → Significant - Specificity: McNemar test χ^2^ = 0.63, *p* = 0.529 → Not significant - Global Wald Test (Se_SE = Se_NSE and Sp_SE = Sp_NSE): χ^2^ = 25.84, *p* < 0.01 - Holm Correction: - Se_SE = Se_NSE: χ2 = 23.02, *p* < 0.001 - Sp_SE = Sp_NSE: χ2 = 0.63, *p* = 0.529						

Abbreviations: US = ultrasound; Se = sensitivity; Sp = specificity; CI = confidence interval. Note: Confidence intervals for the difference in proportions are not statistically significant if they include zero.

**Table 2:** Sensitivity and specificity results of E and NE—joint swelling; 1463 joints.

	Standardized Exam (SE)			Non-Standardized Exam (NSE)		
	Positive	Negative	Total	Positive	Negative	Total
Ultrasound Positive	221	141	362	15	62	77
Ultrasound Negative	73	177	250	84	646	730
Total	294	318	612	99	708	1419
Diagnostic Accuracy—Before and After Adjustment for Reference Standard Bias						
Parameter	Standardized Exam (%)			Non-Standardized Exam (%)		95% CI for Difference
Sensitivity	82.4 (95% CI: 78.6–85.7) → 91.9 (adjusted)			53.7 (95% CI: 49.0–58.3) → 60.0 (adjusted)		(0.29; 0.34)
Specificity	74.4 (95% CI: 71.6–77.1) → 77.1 (adjusted)			83.9 (95% CI: 81.5–86.1) → 85.7 (adjusted)		(–0.11; –0.05)
Statistical Comparison (SE vs. NSE) Before Adjustment for Reference Standard Bias						
- Sensitivity: McNemar test χ^2^ = 100.16, *p* < 0.001 → Significant - Specificity: McNemar test χ^2^ = 32.4, *p* < 0.001 → Significant - Global Wald Test (Se_SE = Se_NSE and Sp_SE = Sp_NSE): χ^2^ = 166.78, *p* < 0.001 - Holm Correction: - Se_SE = Se_NSE: χ^2^ = 134.0, *p* < 0.001 - Sp_SE = Sp_NSE: χ^2^ = 39.7, *p* < 0.001						

Abbreviations: US = ultrasound; Se = sensitivity; Sp = specificity; CI = confidence interval. Note: Confidence intervals for the difference in proportions are not statistically significant if they include zero.

**Table 3:** Comparison of predictive values for joint tenderness according to prevalence and global Wald test.

Prevalence (%)	PPV SE (95% CI)	PPV NSE (95% CI)	NPV SE (95% CI)	NPV NSE (95% CI)	Wald Test (*p*-Value)
10	30.7% (27.3–34.4)	28.0% (24.5–31.8)	96.9% (95.5–97.9)	94.5% (92.8–95.8)	3.518 (*p* = 0.172)
20	47.0% (43.2–50.8)	43.8% (39.7–47.9)	94.1% (92.2–95.5)	89.5% (87.4–91.4)	11.750 (*p* = 0.003)
30	58.4% (54.2–62.4)	55.8% (51.7–60.0)	89.7% (87.6–91.7)	80.1% (77.2–82.8)	23.456 (*p* < 0.001)
40	63.9% (60.2–67.6)	60.9% (56.8–64.8)	88.8% (86.4–90.8)	81.1% (78.4–83.5)	40.355 (*p* < 0.001)
50	68.9% (65.3–72.4)	66.1% (62.1–69.8)	86.4% (83.8–88.6)	77.4% (74.5–80.0)	57.519 (*p* < 0.001)
60	72.7% (69.1–76.0)	70.0% (66.1–73.6)	84.1% (81.4–86.4)	74.0% (71.1–76.8)	74.872 (*p* < 0.001)
70	75.6% (72.2–78.8)	73.1% (69.4–76.6)	81.9% (79.1–84.4)	71.0% (67.9–73.9)	91.654 (*p* < 0.001)
80	78.0% (74.7–81.0)	75.7% (72.0–79.0)	79.8% (76.9–82.5)	68.1% (65.0–71.1)	107.408 (*p* < 0.001)
90	80.0% (76.7–82.9)	77.8% (74.2–81.0)	77.9% (74.9–80.6)	65.5% (62.4–68.6)	121.888 (*p* < 0.001)

PPV: positive predictive value. NPV: negative predictive value. SE: standardized examination. NSE: non-standardized examination. CI: confidence interval.

**Table 4:** Comparison of predictive values for joint Swelling according to prevalence and global Wald test.

Prevalence (%)	PPV SE (95% CI)	PPV NSE (95% CI)	NPV SE (95% CI)	NPV NSE (95% CI)	Wald Test (*p*-Value)
10	31.9 (28.3–35.7)	32.7 (28.2–37.5)	96.7 (95.2–97.7)	92.6 (90.9–94.1)	22.300 (*p* < 0.001)
20	48.3 (44.4–52.3)	49.3 (44.4–54.2)	93.6 (91.7–95.1)	86.2 (84.0–88.2)	69.400 (*p* < 0.001)
30	59.2 (55.2–63.0)	60.1 (55.1–64.8)	90.5 (88.2–92.3)	80.2 (77.7–82.5)	131.200 (*p* < 0.001)
40	65.2 (61.3–68.9)	66.0 (61.2–70.5)	88.0 (85.6–90.1)	75.8 (73.1–78.3)	183.400 (*p* < 0.001)
50	71.0 (67.0–74.9)	72.1 (67.3–76.5)	85.2 (82.3–87.8)	72.5 (69.4–75.4)	245.700 (*p* < 0.001)
60	73.7 (70.1–77.1)	74.5 (69.9–78.5)	83.0 (80.3–85.5)	67.6 (64.7–70.4)	288.508 (*p* < 0.001)
70	76.6 (73.1–79.8)	77.3 (72.9–81.2)	80.7 (77.9–83.3)	64.1 (61.2–67.1)	333.072 (*p* < 0.001)
80	78.9 (75.5–82.0)	79.5 (75.3–83.3)	78.6 (75.6–81.3)	61.1 (58.0–64.0)	371.843 (*p* < 0.001)
90	80.8 (77.5–83.8)	81.4 (77.3–84.9)	76.5 (73.5–79.3)	58.2 (55.2–61.2)	405.013 (*p* < 0.001)

PPV: positive predictive value. NPV: negative predictive value. SE: standardized examination. NSE: non-standardized examination. CI: confidence interval.

**Table 5:** Comparison of predictive values before and after bias correction.

Condition	PV Type	Exam SE Before (%)	Exam SE After (%)	Exam NSE Before (%)	Exam NSE After (%)	95% CI for Difference * (SE–NSE)
Joint Tenderness	PPV	57.2	88.4	54.0	72.8	(0.12; 0.18)
	NPV	91.3	96.8	84.9	89.4	(0.10; 0.14)
Joint Swelling	PPV	59.2	87.1	60.1	83.7	(0.27; 0.33)
	NPV	90.5	95.8	80.2	83.7	(0.09; 0.14)

PV: predictive value. PPV: positive predictive value. NPV: negative predictive value. SE: standardized examination. NSE: non-standardized examination. CI: confidence interval. * Note: The confidence interval for the difference in proportions is not statistically significant if it includes zero.

## Data Availability

Data available in a publicly accessible repository: The original data presented in the study are openly available in FigShare at DOI: 10.6084/m9.figshare.29211236.
